# The Spirit of Sacrifice and Service

**Published:** 1920-06-26

**Authors:** 


### ASSOCIATION OF HOSPITAL MATRONS.

### The Spirit of Sacrifice and Service.

The annual meeting of the above Association was held at St. Thomas's Hospital, on Saturday, June 19. The chair was taken by the President, Miss Lloyd Still, C.B.E., R.R.C., and some 210 members were present.

In her opening remarks the President expressed regret at the death during the past year of two most loyal friends of the nursing profession, Sir Henry Burdett and Sir Robert Morant. She reminded the members that while a year ago the Association barely existed, to-day it was an organised body of professional women, leaders of a profession responsible for the well-being of the women who are to be the nurses of the future. When speaking of the nursing profession as not being what it should be, or what it was, it must be remembered that nurses are, in a great measure, influenced by the tone of the hospital in which they work. A strong body must be built up of well-trained, conscientious, skilled women, ready to face the strain of the work which was before them. The Association must confer together on the many problems confronting the profession—education, hours of employment or shortened hours, the difficulties of small and special hospitals, methods of affiliation—all these with sympathetic consideration of the needs of the sick, which in some cases in the excitement of the moment are lost sight of. The President urged that it was their privilege and responsibility to check in their ranks the prevalent idea in the working world that work is drudgery. While not condoning overwork, she averred that, if the profession is to maintain its true life, the spirit of sacrifice and service must be maintained. After drawing attention to the increased general recognition of the Association as a body whose considered opinion was counted to be of value, the President concluded her remarks by paying a tribute to the Honorary Secretary and Honorary Treasurer, whose work has been the mainstay of the Association.

The Honorary Secretary, Miss Cox Davies, R.R.C. (Royal Free Hospital), announced the receipt of a large number of letters of regret for non-attendance, and drew attention to one from Miss Scott (Sussex County Hospital, Brighton), inviting the Association to hold its next meeting at Brighton. The annual report was then read, containing particulars of the activities of the Association which, while only founded in May 1919, had now reached a total membership of no less than 367, made up as follows: General hospitals over 100 beds, 76 members; general hospitals under 100 beds, 59 members; special hospitals, 104 members; Poor-law infirmaries, 35 members; Military hospitals, 25 members; Superintendents of nursing institutions, 41; late matrons, 27.

The financial report was read by the honorary treasurer, Miss Finch (University College Hospital), and both reports were passed unanimously by the meeting.

A resolution, proposed by Miss Musson and seconded by Miss Dalton, "That in future the quarterly meetings be held in October, January and April, the annual meeting to take place in June," was discussed and carried, it being understood that the annual meeting in June, 1921, should be arranged so that it should not clash with the College of Nursing meeting, also fixed for that month.

A discussion then took place on the formation of Local Groups of the Association in various areas, and Miss Monk, in moving an amendment to the Constitution of the Association to carry this into effect, pointed out that local groups would be more representative and more practical than the central body, which was far away in an emergency. The amendment was seconded by Miss Barton, and carried unanimously. Reports from four local groups already established were read, from Manchester (Miss Sparshott), Birmingham (Miss Bodley), Winchester (Miss Carpenter Turner), and South Devon and Cornwall (Miss Chaff), on which the President said she thought it had been seen that the branches justified their existence, and notified the meeting that applications for the formation of branches would be considered. She thought it was the only way to keep the Association alive; the branches were the children of the Association, and must be in touch with it.

The President and the Honorary Officers of the Association were accorded a hearty vote of thanks, and the proceedings then terminated.

